# The histone deacetylase inhibitor sodium valproate causes limited transcriptional change in mouse embryonic stem cells but selectively overrides Polycomb-mediated *Hoxb* silencing

**DOI:** 10.1186/1756-8935-6-11

**Published:** 2013-05-01

**Authors:** Elsa Boudadi, Hannah Stower, John A Halsall, Charlotte E Rutledge, Martin Leeb, Anton Wutz, Laura P O’Neill, Karl P Nightingale, Bryan M Turner

**Affiliations:** 1 Chromatin and Gene Expression Group, College of Medical and Dental Sciences, Institute of Biomedical Research, University of Birmingham, Birmingham B15 2TT, UK; 2 Wellcome Trust - Medical Research Council Stem Cell Institute, University of Cambridge, Tennis Court Road, Cambridge, CB2 1QN, UK

**Keywords:** *Hoxb* genes, Valproic acid, Histone deacetylase, Polycomb repression, Mouse embryonic stem cells, Histone modification, Microarray expression analysis, Retinoic acid, Transcriptional activation

## Abstract

**Background:**

Histone deacetylase inhibitors (HDACi) cause histone hyperacetylation and H3K4 hypermethylation in various cell types. They find clinical application as anti-epileptics and chemotherapeutic agents, but the pathways through which they operate remain unclear. Surprisingly, changes in gene expression caused by HDACi are often limited in extent and can be positive or negative. Here we have explored the ability of the clinically important HDACi valproic acid (VPA) to alter histone modification and gene expression, both globally and at specific genes, in mouse embryonic stem (ES) cells.

**Results:**

Microarray expression analysis of ES cells exposed to VPA (1 mM, 8 h), showed that only 2.4% of genes showed a significant, >1.5-fold transcriptional change. Of these, 33% were down-regulated. There was no correlation between gene expression and VPA-induced changes in histone acetylation or H3K4 methylation at gene promoters, which were usually minimal. In contrast, all *Hoxb* genes showed increased levels of H3K9ac after exposure to VPA, but much less change in other modifications showing bulk increases. VPA-induced changes were lost within 24 h of inhibitor removal. VPA significantly increased the low transcription of *Hoxb4* and *Hoxb7*, but not other *Hoxb* genes. Expression of *Hoxb* genes increased in ES cells lacking functional Polycomb silencing complexes PRC1 and PRC2. Surprisingly, VPA caused no further increase in *Hoxb* transcription in these cells, except for *Hoxb1*, whose expression increased several fold. Retinoic acid (RA) increased transcription of all *Hoxb* genes in differentiating ES cells within 24 h, but thereafter transcription remained the same, increased progressively or fell progressively in a locus-specific manner.

**Conclusions:**

*Hoxb* genes in ES cells are unusual in being sensitive to VPA, with effects on both cluster-wide and locus-specific processes. VPA increases H3K9ac at all *Hoxb* loci but significantly overrides PRC-mediated silencing only at *Hoxb4* and *Hoxb7. Hoxb1* is the only *Hoxb* gene that is further up-regulated by VPA in PRC-deficient cells. Our results demonstrate that VPA can exert both cluster-wide and locus-specific effects on *Hoxb* regulation.

## Background

Histone deacetylase inhibitors (HDACi) have long been known to cause global histone hyperacetylation, often accompanied by increased H3K4 methylation, in a variety of model systems ([1] and references therein). Two structurally unrelated HDACi, suberoylanilide hydroxamic acid (SAHA) and depsipeptide (a bicyclic peptide) are remarkably effective against cutaneous T-cell lymphoma (CTCL) [2,3] and have been Food and Drug Administration (FDA) approved for treatment of this cancer (Additional file 1: Table S1). HDACi have great potential as chemotherapeutic agents, prompting searches for new HDACi and a growing number of trials against various cancers [4,5]. A major barrier to improving the clinical effectiveness of HDACi is that their mechanisms of action are varied and complex, and generally not well understood (discussed in [6]). There are at least six different structural classes of HDACi, four of which are in clinical trials (Additional file 1: Table S1). All exert multiple effects on cell function, including induction of differentiation, cell cycle disruption and apoptotic death [5,6]. The situation is further complicated by the fact that there are 18 different histone deacetylases (HDACs) in human cells, split into four classes [5,7]. Eleven of these enzymes, classes I, IIa, IIb and IV, have a very similar catalytic site, but differ in subtle ways in their sensitivities to HDACi (Additional file 1: Table S1) [6]. Class III enzymes, the sirtuins, are NAD-dependent and are insensitive to all classes of HDACi in clinical use [8]. In addition, HDACs, despite their name, act on a variety of proteins in addition to histones [9], including transcription factors, enzymes and HDACs themselves [10]. They usually operate *in vivo* as part of multi-protein complexes, the composition of which can influence their catalytic activity, their location within the cell and their targeting to specific genes [7,9].

Valproic acid (VPA) is a branched, short-chain fatty acid that inhibits class I and IIa HDACs, most likely through binding to the catalytic site [11]. VPA has been used clinically for many years as an anti-epileptic agent and mood stabiliser, usually as the sodium salt [11,12]. Because it is well tolerated and has been shown to induce differentiation and apoptosis of carcinoma cells, it has recently been tested in clinical trials as a potential chemotherapeutic agent for a variety of cancers [4,13]. One long-appreciated side effect of VPA is its teratogenicity, causing problems for those women who must depend on it during pregnancy because alternative drugs are ineffective or unavailable [14,15]. Teratogenic effects include musculoskeletal, neurological and behavioural aberrations, leading to identification of a distinctive valproate syndrome [15-17]. VPA acts on class I and IIa HDACs, which collectively deacetylate a variety of nuclear and cytoplasmic proteins, so its effects are inevitably pleiotropic. For example, VPA induces oxidative stress, with increased levels of reactive oxygen species, and anti-oxidants can alleviate some of its teratogenic effects [18,19].

Surprisingly, global changes in histone modification induced by HDACi are usually not accompanied by changed levels at individual genes, as measured by chromatin immunoprecipitation (ChIP). In fact, butyrate has been reported to cause an unexpected decrease in histone acetylation at some transcription start sites in HepG2 and HT29 cells, with associated down-regulation of transcription [20]. A wide ranging ChIP-seq study of quiescent human T-cells showed that a combination of butyrate and Trichostatin A (TSA) at high concentrations induced acetylation primarily at the promoters of active genes and of the small proportion of silent genes that showed high levels of H3K4 methylation [21]. In human lymphoblastoid cells we found that only a small proportion of genes showed altered transcription after treatment with VPA. Of these, almost as many were down-regulated as were up-regulated [22], consistent with early findings of a surprising lack of transcriptional stimulation by HDACi [23]. Nor did we find any consistent increase in histone acetylation or H3K4 methylation at selected promoter regions, leading to the suggestion that many genes are sheltered from the global effects of HDACi [22].

The complex relationship between the histone modifications induced by HDACi and the transcription of individual genes reflects the general uncertainty surrounding the role of histone modifications in transcriptional control. The recent achievements in genome-wide mapping of chromatin marks have confirmed that some histone modifications are usually associated with transcriptionally active, or potentially active, genes (for example, H3K9ac, H3K4me3), while others are typical of silent genes (for example, H3K9me2/3, H3K27me3) [24,25]. However, such data cannot establish the cause-effect relationship between histone modification and gene activity. It is important to establish for particular loci in particular developmental contexts, whether a histone modification can by itself drive a change in chromatin function, whether the modification is part of a chain of events by which chromatin transitions from one functional state to another, or whether the modification is simply a by-product of other processes and of no functional consequence in its own right [26,27].

In the experiments reported here, we have tested the ability of VPA to alter patterns of gene expression in mouse embryonic stem (ES) cells, looking in detail at its effects on members of the *Hoxb* cluster. The *Hox* (*Homeobox*) genes encode highly conserved transcription factors that act as master regulators of development and whose mis-expression has been implicated in a variety of human diseases, including cancer [28,29], and possibly in the teratogenicity of VPA [15]. In vertebrates, 39 *Hox* genes are divided between four clusters (A, B, C and D) located on different chromosomes. They are silent or very weakly expressed in the pre-implantation mammalian embryo and in ES cells, but become activated during development along the antero-posterior axis according to their positions in the cluster [30]. Previous work has indicated that the chromatin that packages the *Hoxb* genes in embryonic cells can be influenced by HDAC inhibitors [31,32]. Here, we use them as a model system to explore the relationship between changes in histone modifications and transcription as genes switch from silenced to active states.

## Results

### VPA causes global histone modification changes in ES cells but has little effect on gene expression or differentiation state

**Figure 1:**
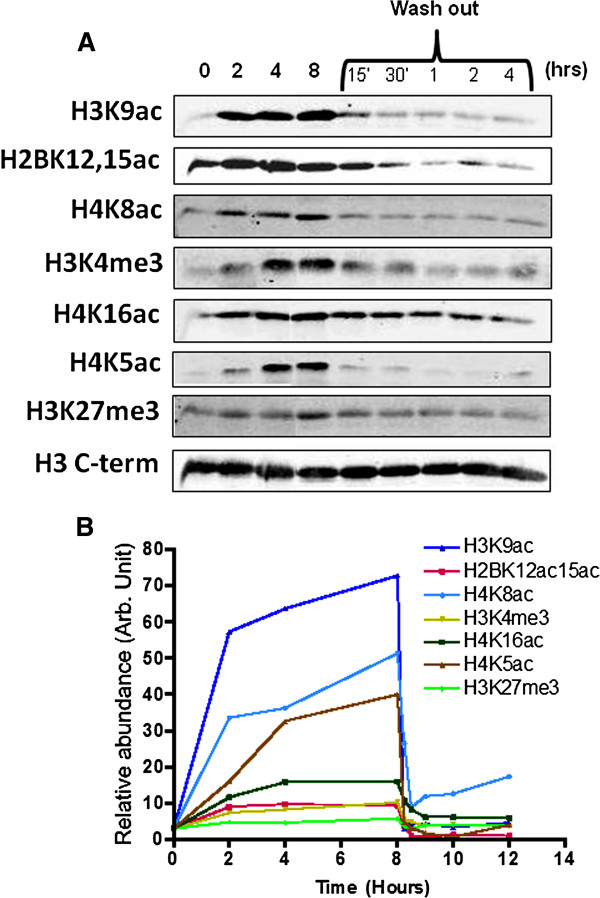
**Valproic acid induces global histone hyperacetylation and H3K4 hypermethylation in embryonic stem cells. **(**A**) Western blots showing changes in the indicated histone modifications after treatment of embryonic stem (ES) cells (CCE/R) for up to 8 h with 1 mM valproic acid (VPA), and at various timepoints after its removal (wash out). Staining with an antibody to the unmodified H3 C-terminal domain was used as a loading control (H3 C-term, bottom tracks). (**B**) Measurement of the fluorescent secondary antibody signal quantifies changes in histone modifications over time (n = 3).

ES cells treated with 1 mM VPA for up to 8 h showed a progressive increase in acetylation of H2B, H3 and H4 and in trimethylation of H3K4, all of which began to level off after 2 to 4 h (Figure 1). The induced increases were rapidly reversible, returning to pre-treatment levels or below within less than 30 minutes of removal of the inhibitor (Figure 1). This behaviour is consistent with what has been seen in other cell types ([1] and references therein).

**Figure 2:**
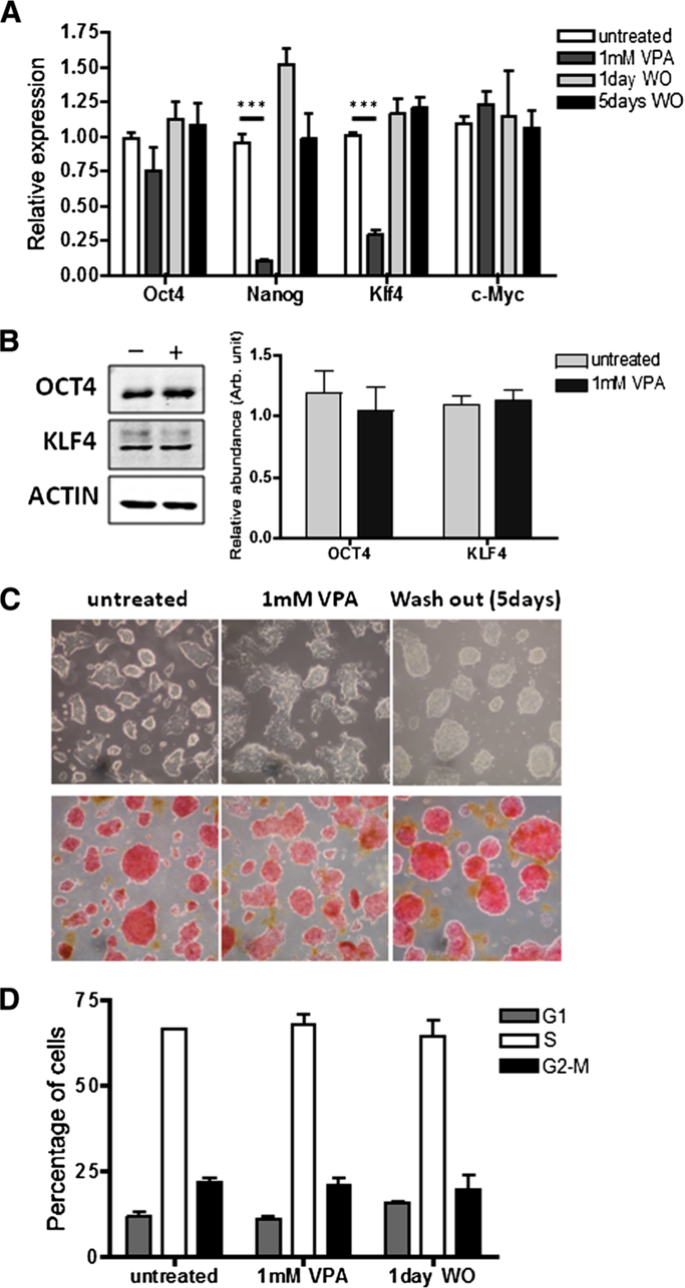
**Valproic acid reduces expression of selected pluripotency genes but does not trigger long-term changes in cell growth or differentiation. **(**A**) Embryonic stem (ES) cells (CCE/R) were treated for 8 h with 1 mM valproic acid (VPA) followed by culture for one or five days in the absence of the drug (wash out, WO). The expression of four pluripotency-associated genes was quantified by real time-quantitative (RT-q)PCR, relative to the expression of β-actin, in untreated, VPA-treated and WO cells. Error bars represent the standard error (SE) of the mean of independent experiments (n = 3). To assess the significance of a change, two-tailed unpaired *t*-tests were performed using GraphPad software; ***P < 0.001. (**B**) Levels of OCT4 and KLF4 proteins were checked before (left lane) and after (right lane) VPA treatment by western blotting. A measure of the secondary antibody fluorescent signal, normalised to the β-actin signal, allowed a quantitative representation of changes. Error bars indicate SE from 3 independent experiments. (**C**) Light microscopy was used to assess the cell morphology at each timepoint (upper panels). Alkaline phosphatase activity (red staining) was used as a marker of pluripotency (lower panels, pictures taken using 20× magnification). (**D**) ES cells were stained with propidium iodide (PI) and analysed by flow cytometry to obtain the mean percentage of cells in each phase of the cell cycle before and after VPA treatment and WO (n = 3).

The VPA treatment caused a several-fold drop in transcripts from the pluripotency genes *Nanog* and *Klf4*, a slight drop in expression of *Oct4* (a marker of undifferentiated cells) but no significant change in *cMyc* (Figure 2A). OCT4 and KLF4 proteins were not affected over the timescale of the experiment (Figure 2B; we were unable to detect NANOG protein by western blotting). Importantly, expression of these genes was restored within 1 day of removal of VPA (Figure 2A). There was a consistent change in morphology after 8 h in VPA, with cells tending to become more flattened. However, this change was not accompanied by loss of pluripotency, as shown by alkaline phosphatase (AP) staining, and morphology returned to normal following VPA removal (Figure 2C). Thus, we find no evidence that treatment of ES cells with VPA for up to 8 h permanently disturbs their undifferentiated state. Over an 8-h treatment time, 1 mM VPA did not detectably change the cells’ characteristic cell cycle profile, with more than 60% of cells in S-phase in both treated and untreated cultures (Figure 2D), nor did it increase the number of dead or metabolically inactive cells.

**Figure 3:**
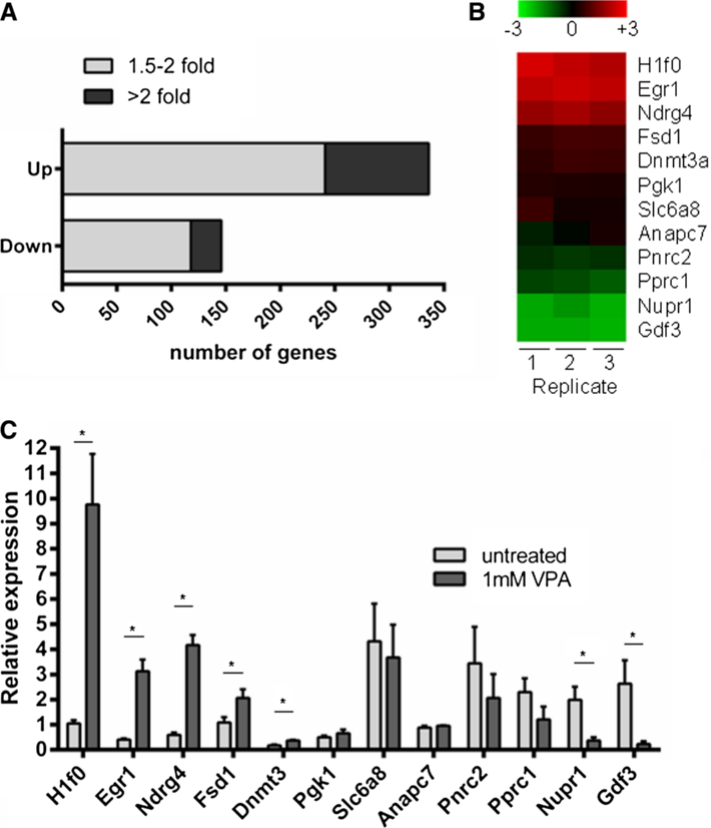
**Valproic acid treatment changes expression of only a small proportion of genes in embryonic stem cells. **Embryonic stem (ES) cells (CCE/R) were treated for 8 h with 1 mM valproic acid (VPA) and the NIA15K cDNA microarray was used to compare gene expression in control and treated cells. The use of three biological replicates allowed statistical testing (*t*-tests) to classify genes by the level of significance (*P-*value) of their expression change. (**A**) Genes for which the change in transcript level (up or down) had a *P-*value after FDR correction, equal to or less than 0.1, and a mean change of at least 1.5-fold, were classed as up- or down-regulated, as indicated. The histogram shows the numbers of genes where the significant fold change is 1.5- to 2.0-fold (light grey) or >2.0-fold (dark grey). (**B**) Heat map showing gene expression change in three replicate experiments along with a list of representative genes showing different levels of change. (**C**) To validate microarray results, representative transcripts were quantified by real time-quantitative (RT-q)PCR in untreated and VPA-treated cells. Error bars represent the standard error of the mean of independent experiments (n = 3). To assess significance of a change, two-tailed unpaired *t*-tests were performed using the GraphPad software; *P <0.05*.*

To compare global transcription levels in control and VPA-treated ES cells, we used the NIA15K array, comprising 15,247 unique oligo(dT)-primed cDNA clones derived from 52,374 Expressed Sequence Tags (3′ ESTs), mostly from early embryonic cDNA libraries [33]. The NIA15K array is ideal for the accurate measurement of gene expression levels in embryonic cells [34,35]. Two-colour (cy3/cy5) labelling was used to compare VPA-treated and untreated samples. M-values, representing the ratio between the cy3 and cy5 fluorescent signals, from three independent experiments were tested for statistical significance using *P-*values corrected using the Benjamini False Discovery Rate (FDR) correction. After 8 h in 1 mM VPA, 1,144 genes (7.5%) on the array showed a significant change in transcription activity (*P* ≤0.1 after FDR correction), of which 674 were up- and 470 down-regulated. However, for many of these genes the fold changes were small and when we applied additional thresholds the number of genes showing changes dropped rapidly. Thus, only 359 genes (2.4%) changed between 1.5- and 2-fold (241 up-, 118 down-regulated), and just 123 (0.8%) changed more than 2-fold (95 up and 28 down). These results are summarised in Figure 3A. Selected genes for which replicate microarray analyses consistently showed differing degrees of VPA-dependent change in expression (Figure 3B), were tested by RT-qPCR. Results were consistent with both the direction and relative magnitude of change detected by microarray analysis; examples are shown in Figure 3C.

Ontology analysis of this dataset, using DAVID software, revealed several annotations to be significantly over-represented amongst genes whose activities were altered by VPA. The most significant are listed in Additional file 1: Table S2. The highest scoring annotation was Cytoskeleton and cytoskeleton-interacting proteins (*P* = 5.13 × 10^-5^). Cell cycle (*P* = 6.76 × 10^-4^) and chromatin organisation (*P* = 6.46 × 10^-3^) were also featured. The effect on cytoskeleton-related genes is intriguing in light of the morphological changes induced by VPA. However the short timescale involved and the reversibility of the effect makes it likely that morphological changes were the result of a combined effect of altered acetylation of cytoskeletal proteins and gene expression changes [36].

### VPA-induced changes in transcription at specific genes are not consistently linked to changes in histone acetylation

**Figure 4:**
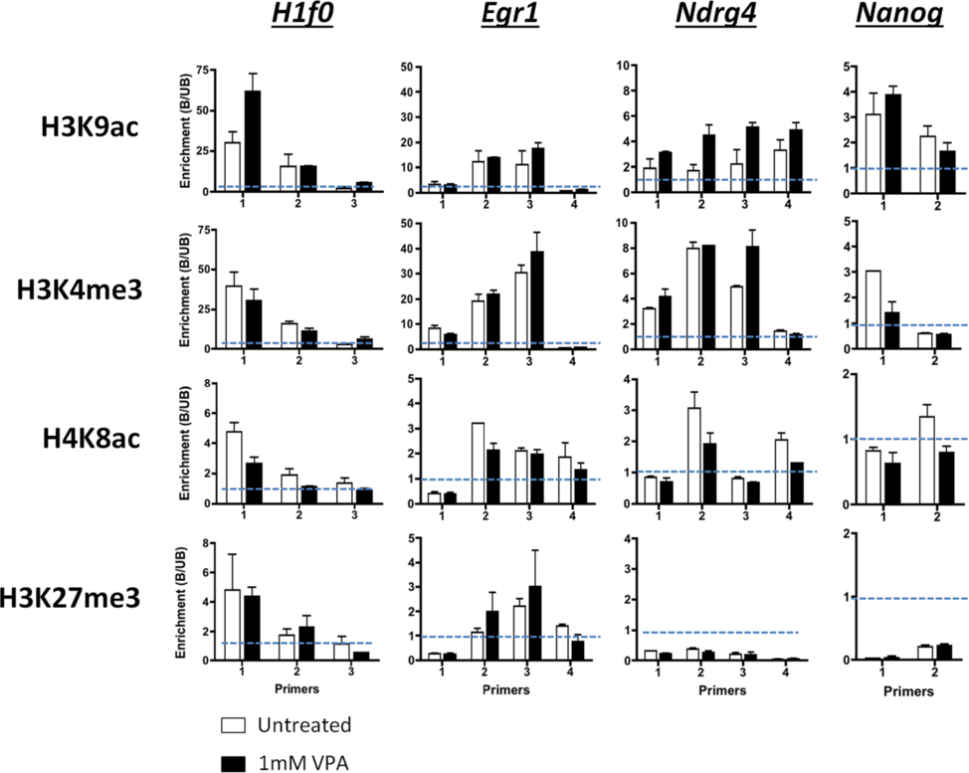
**Valproic acid-induced changes in transcription at specific genes are not consistently linked to changes in histone acetylation. **Modified histones were assayed at selected regions of genes showing increased expression (*H1f0, Egr1, Ndrg4*), or decreased expression (*Nanog*) in response to valproic acid (VPA). Chromatin was prepared from CCE/R embryonic stem (ES) cells before (white bars) and after (black bars) VPA treatment (1 mM, 8 h) and immunoprecipitated with antibodies against H3K9ac, H3K4me3, H4K8ac and H3K27me3, as indicated. DNA extracted from bound (B) and unbound (UB) fractions was analysed by real time-quantitative (RT-q)PCR using region-specific primers (Additional file 1: Table S7 shows both the sequences and positions relative to the transcription start site of each primer pair). Relative enrichment in each modification was calculated as the B/UB ratio; a region can be considered enriched in a particular modification if B/UB exceeds 1.0 (dashed lines). Error bars represent the standard error of the mean of independent experiments (n = 2): apparent absence of error bars occurs where the error is too low to be visible at this scale.

To ask whether these expression changes were associated with changes in histone modification at promoter or intragenic regions, we used ChIP to test genes that showed varying levels of expression change after VPA treatment. Examples (*Egr1*, *H1f0*, *Ndrg4, Nanog*) are shown in Figure 4. None of these genes showed any significant VPA-induced change in H3K27me3, H4K8ac or H3K4me3 (Figure 4) despite the global increase in the latter two modifications induced by VPA (Figure 1). H3K9ac levels were more variable, with occasional modest increases (1.5- to 2-fold) at sites across *Ndrg4* and *H1f0* (Figure 4). However, for this modification too, the ChIP results reflect neither the global changes in histone modification, nor the changes in transcript levels seen in response to VPA (Figures 1 and 3). This lack of correlation between HDACi-induced global and local acetylation, and between acetylation and transcription, is consistent with previous results [20,22,37].

### *Hoxb* genes show increased H3 acetylation following VPA treatment, but this is associated with increased transcription only at certain loci

**Figure 5:**
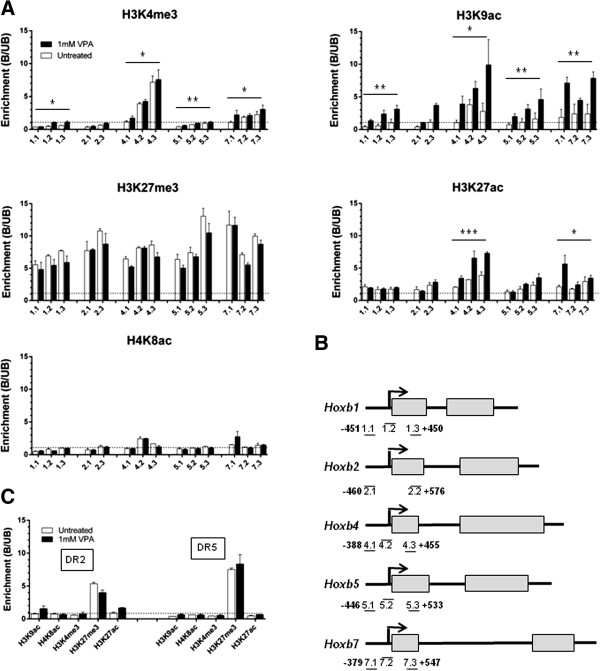
**Valproic acid changes acetylation of some histone lysines at *Hoxb *promoter regions. **(**A**) Levels of H3K9ac, H3K4me3, H3K27me3, H3K27ac and H4K8ac across *Hoxb *genes were assayed by chromatin immunoprecipitation-quantitative PCR (ChIP-qPCR) of chromatin from embryonic stem (ES) cells, either treated with 1 mM valproic acid (VPA) for 8 h (black bars) or untreated (white bars). Regions with bound (B)/unbound (UB) ratios >1.0 (dashed line) can be considered relatively enriched in that modification. Error bars represent the standard error of the mean of independent experiments (n = 3 for H3K27ac, n = 2 for the remainder): apparent absence of error bars occurs where the error is too low to be visible at this scale. (**B**) Positions of *Hoxb *primers (1.1, 1.2 et cetera.) are shown, along with the positions of the most upstream and downstream primer bases, relative to the TSS (-451, +450 etc.). (**C**) Levels of histone modifications across the retinoic acid response elements *DR2 *and *DR5 *assayed by ChIP-qPCR of chromatin from ES cells, either treated with 1 mM VPA for 8 h (black bars) or untreated (white bars).

We previously showed that treatment with VPA of cultured mouse embryos at the 8-cell to morula stage, caused increased acetylation and H3K4 methylation at *Hoxb1* and *Hoxb9*, but not at other genes [32]. In ES cells, we found that treatment with VPA caused a consistent increase in H3K9 acetylation at all *Hoxb* promoter regions, with the level of acetylation increasing from 2-h to 8-h exposure time (Additional file 2: Figure S1, A). Thus, in contrast to its lack of effect on most other genes, VPA increases promoter H3K9 acetylation across the entire *Hoxb* cluster, including *Hoxb13* located about 70 kb from its nearest *Hoxb* neighbour, *Hoxb9* (Additional file 2: Figure S1, C). Subsequent experiments showed that VPA increased H3K9 acetylation at upstream, promoter and intragenic regions of *Hoxb* genes (Figure 5A, B). VPA induced more modest (though sometimes statistically significant) increases in H3K4me3 at these same regions (Figure 5A; see also Additional file 2: Figure S1, B). Surprisingly, H4K8ac was unchanged while increases in H3K27ac were limited to *Hoxb4* and *Hoxb7* (Figure 5A). As expected [38], all *Hoxb* genes showed relatively high levels of H3K27me3, which decreased slightly (though not significantly for any single gene) after VPA treatment (Figure 5A).

In pre-implantation mouse embryos in culture, VPA-induced increases in both histone acetylation and H3K4me3 were heritable through mitosis in the absence of the inhibitor, at least until the blastocyst stage [32]. In contrast, in ES cells the VPA-induced increases in H3K9ac were consistently lost within 24 h of removal of the inhibitor. A typical experiment is shown in Additional file 2: Figure S2. It will be interesting to explore possible reasons for the different behavior of pre-blastocyst embryos and cultured cells (ES cells) derived from the Inner Cell Mass of the blastocyst.

**Figure 6:**
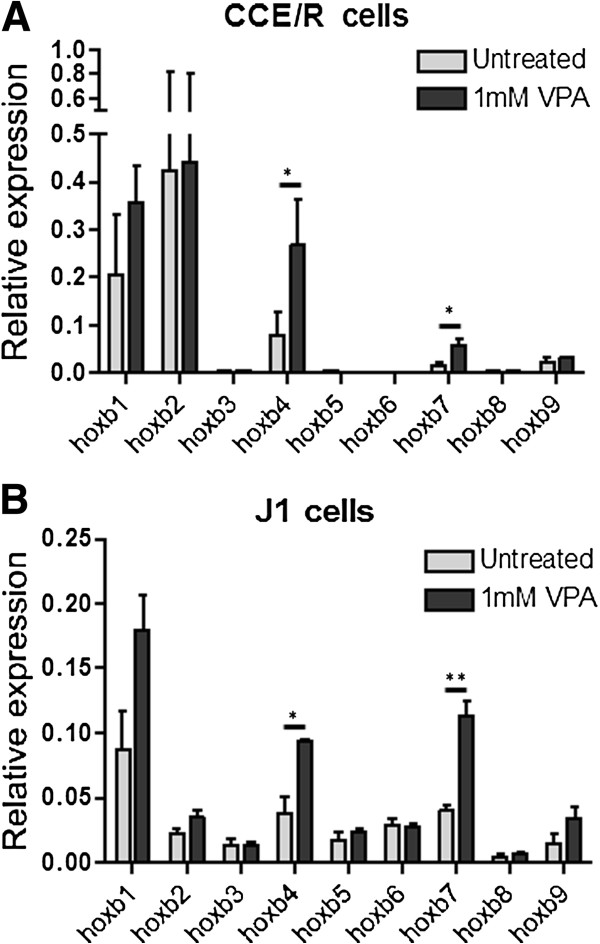
**Valproic acid induces transcription of specific *Hoxb *genes in undifferentiated embryonic stem cells. **Two mouse embryonic stem (ES) cell lines CCE/R (panel **A**, feeder-independent) and J1 (panel **B**, feeder-dependent and the parent line of the *Eed/Ring1b *double knockout (dKO) cells) were treated with valproic acid (VPA) (1 mM, 8 h) and harvested, along with untreated controls, for preparation of RNA and cDNA. Transcript levels from *Hoxb1-9* were determined by real time-quantitative (RT-q)PCR. Error bars show the standard error of the mean of independent experiments (n = 3). Two-tailed unpaired *t*-tests were used to assess significance of changes; *P <0.05, **P <0.01.

To ask whether VPA could change the expression of ES cell *Hoxb* genes we tested two lines, CCE/R (feeder-independent) and J1 (feeder-dependent and parent line of the double knockout (dKO) cells used later) using RT-qPCR. With the single exception of *Hoxb2*, transcript levels before treatment were similar in the two lines. The effect of VPA treatment was also very similar in the two lines, with only *Hoxb4* and *Hoxb7* significantly affected, each showing a 2- to 3-fold increase (Figure 6). In untreated CCE/R cells, *Hoxb4* and *Hoxb7* showed the highest levels of H3K4me3, H3K9ac and H3K27ac at selected sites in their promoter regions and also showed the strongest VPA-induced increases in H3K27ac (Figure 5).

In view of these findings, it is interesting to note that of three genes showing strongly increased transcription after VPA (Figure 3C), two showed relatively high levels of H3K27me3 (*H1f0*, *Egr1*), while one (*Ndrg4*) did not (Figure 4). Thus, Polycomb-mediated silencing is not a prerequisite for VPA-induced increases in either transcription or H3K9ac and cannot, in itself, explain the characteristic and unusual response of *Hoxb* genes to this inhibitor.

### *Hoxb* genes are all induced by retinoic acid in differentiating ES cells but longer-term expression varies from gene to gene

**Figure 7:**
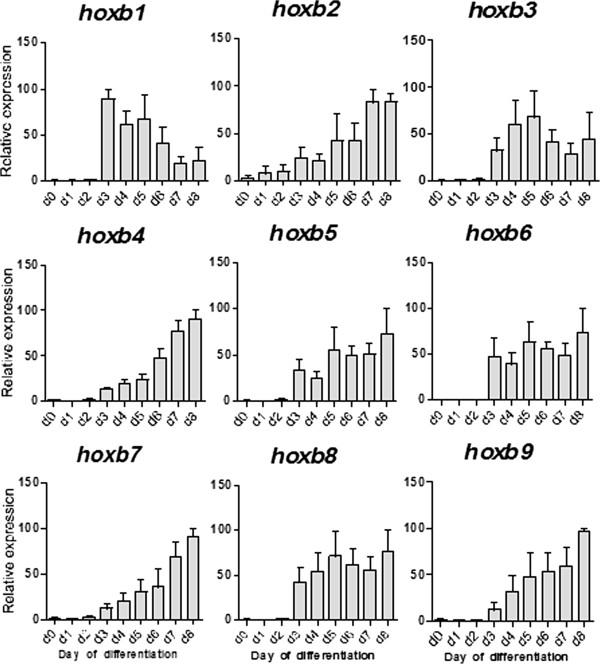
**The timing of *Hoxb *gene expression in mouse embryonic stem cells following induction of differentiation.** Mouse embryonic stem (ES) cells (CCE/R) were induced to differentiate by removal of the growth factor leukaemia inhibitory factor (LIF) and replating at day 0, followed by addition of retinoic acid (RA, 1 μM) at day 2. RNA and cDNA were prepared from cell pellets at day 0 and daily thereafter and transcript levels from individual *Hoxb *genes (*Hoxb *1 to 9) were determined by real time-quantitative (RT-q)PCR. At each timepoint, expression was calculated as a percentage of the highest value in the untreated sample. Error bars represent the standard error of the mean of independent experiments (n = 3).

**Figure 8:**
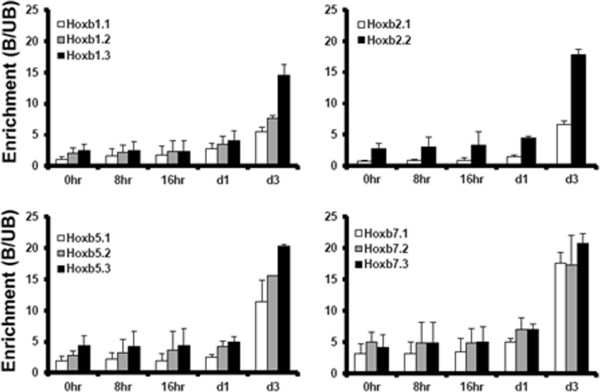
**Treatment of differentiating embryonic stem cells with retinoic acid increases H3K9ac at *Hoxb *genes. **Chromatin immunoprecipitation-quantitative PCR (ChIP-qPCR) was used to investigate H3K9ac levels at two or three regions across four *Hoxb *genes, as indicated, in differentiating embryonic stem (ES) cells (CCE/R) before and after treatment with retinoic acid (RA). Leukaemia inhibitory factor (LIF) was removed at d0 (0 h) and RA added at d2. Location of the primer pairs used is shown in Figure 5B. Data are displayed as the enrichment of the *Hoxb* sequences in the bound relative to the unbound (B/UB) sample for each timepoint. Error bars represent the standard error of the mean of independent experiments (n = 2).

We have used the *Hoxb* gene cluster to explore the relationship between induced changes in histone modification and switching from a silent to an expressed state. Induction of differentiation by removal of leukaemia inhibitory factor (LIF) (day 0), and addition of retinoic acid (RA, day 2), resulted in the rapid up-regulation of all *Hoxb* genes (Figure 7). However, the activation progressed differently depending on the *Hoxb* locus involved. For *Hoxb1, 3, 6* and *8,* expression increased rapidly on day 3 and remained approximately constant, or declined (*Hoxb1* only), whereas *Hoxb2, 4, 5, 7* and *9* all increased slowly, to reach maximum expression around day 8, the latest time point examined. There was no clear correlation between the timing of maximum RA-induced expression of individual *Hoxb* genes and their position in the cluster. Nor was there any correlation between the levels of histone modification tested at *Hoxb* promoters (Figure 5) and the longer-term progression of the RA-induced increase in transcription. Following RA treatment (day 2) there was by day 3 a several-fold increase in H3K9ac at promoter-proximal and coding regions of all *Hoxb* genes tested (*Hoxb1*, *2*, *4*, *5*, *7* and *9*). Illustrative results are shown in Figure 8. In view of previous results, it is significant that induction of ES cell differentiation by LIF withdrawal increased neither *Hoxb* transcription nor H3K9ac levels at *Hoxb* promoters (Figures 7 and 8). Thus, the short-term effects of VPA on H3K9ac at *Hoxb* promoters and *Hoxb* expression, cannot be attributed to induction of differentiation in a subpopulation of ES cells.

### Loss of Polycomb components generally increases *Hoxb* gene expression and modulates the transcriptional response to VPA in a locus-specific manner

To test the role of Polycomb in silencing *Hoxb* in undifferentiated ES cells, we measured expression of *Hoxb* genes in an ES cell line in which both PRC1 and PRC2 were non-functional [39]. Total lack of expression of *Ring1b* (PRC1) and *Eed* (PRC2) in these dKO cells was confirmed by qPCR (Additional file 2: Figure S3). Loss of Polycomb components in those cells led to a strong diminution of the H3K27me3 mark, as expected, and a corresponding increase in global H3K27ac (Additional file 2: Figure S3).

**Figure 9:**
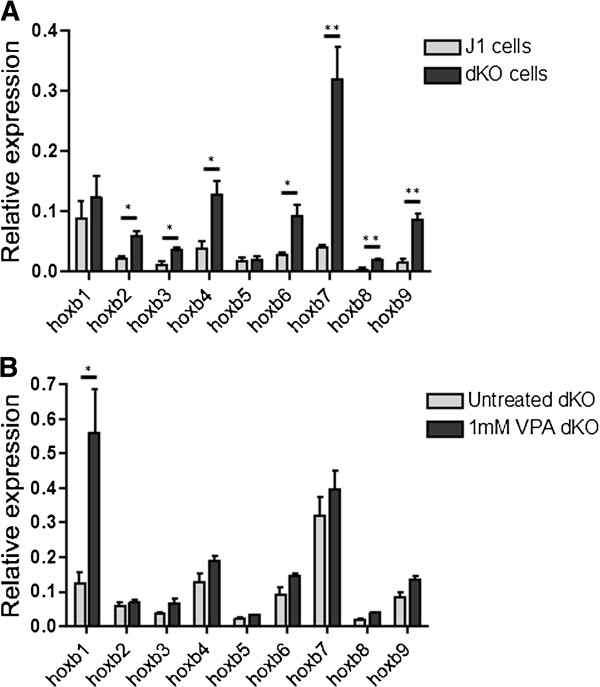
**Knockout of Eed/Ring1b increases expression of *Hoxb *genes but only *Hoxb1 *responds additionally to valproic acid. **(**A**) Transcript levels of *Hoxb *genes were quantified by real time-quantitative (RT-q)PCR in wild-type embryonic stem (ES) cells (J1 line) and in J1 cells in which two genes encoding Polycomb proteins (*Eed*; *Ring1b*) had been knocked out (dKO cells). Light grey bars show the expression levels in wild-type J1 cells, and dark grey bars represent expression in dKO cells. (**B**) dKO cells were treated for 8 h with valproic acid (VPA) (1 mM). *Hoxb *transcript levels in treated and untreated cells were assayed by RT-qPCR. Light grey bars represent untreated dKO cells, dark grey bars show data for VPA-treated cells. For both panels error bars represent the standard error of the mean of independent experiments (n = 3). Two-tailed unpaired *t*-tests were used to assess the significance of differences; *P <0.05*,* **P <0.01.

*Hoxb* genes, showed a several-fold increase in expression in dKO cells (Figure 9A), with the exception of *Hoxb5*, whose activity remained very low, and possibly *Hoxb1* (where the increase was not significant). However, even in the absence of Polycomb, levels remained well below those observed after differentiation and exposure to RA (Figure 7). As in wild-type cells, treatment of dKO cells for 8 h with 1 mM VPA induced global hyperacetylation of histones and a modest increase of H3K4me3 (Additional file 2: Figure S3). However, with the exception of *Hoxb1*, VPA caused no further increase in the expression of *Hoxb* genes over that induced by loss of Polycomb (Figure 9B). *Hoxb1* was a clear exception, showing a 4.5-fold increase in expression after VPA treatment.

The comparatively weak activation of *Hoxb1* in response to Polycomb depletion (Figure 9A), its strong response to VPA in Polycomb null cells (Figure 9B) and the unique diminution of RA-induced *Hoxb1* expression in differentiating cells over time (Figure 7), all suggest that *Hoxb1* is subject to a regulatory regime that differs from other *Hoxb* genes. *Hoxb1* is distinguished by its proximity to two RA response elements (RAREs), DR2 and DR5, which lie 1.5Kb and 6.5Kb respectively, 3′ of the *Hoxb1* coding region. We find both RAREs in CCE/R ES cells are strongly enriched in the silencing mark H3K27me3, with below average levels of H3K27ac, H3K9ac, H4K8ac and H3K4me3 (Figure 5C). VPA treatment caused no changes in the modifications tested at DR5, but DR2 showed an approximately 2-fold increase in H3K9ac and H3K27ac, along with a small diminution of H3K27me3 (Figure 5C). These changes, though modest, may contribute to the distinctive transcriptional responses of *Hoxb1*.

## Discussion

ES cells treated with VPA showed dramatic global changes in histone modification, including increased acetylation of all histones and H3K4 methylation, but surprisingly limited changes in both transcript levels and histone modification levels at individual promoters. In line with the limited transcriptional response, we found only very occasional evidence for genes showing altered histone acetylation or H3K4 methylation following growth in VPA, even among those genes showing the strongest changes in transcription. This discrepancy between global hypermodification and minimal local modification changes is consistent with our recent studies on human lymphoblastoid cell lines [22], and suggests that in ES cells, as in other cell types, many genes are sheltered from the global changes in histone modification induced by environmental inhibitors. This may also help explain the low frequency of VPA-induced changes in gene expression. Following completion of the microarray experiments presented here, Hezroni and colleagues [40] published data showing limited transcriptional change following treatment of ES cells with VPA. Comparison of our data with the nearest equivalent set from Hezroni and colleagues, shows a strong overlap, despite differences in treatment time and concentration (0.5 mM VPA for 4 h vs 1 mM for 8 h). Of our 241 up-regulated (>1.5-fold) genes, 52 were classified as up-regulated by Hezroni *et al*. [40]. The overlap for down-regulated genes was less striking at 118 and 18 respectively. Genes in common are listed in Additional file 1: Table S3. Ontology analysis (DAVID) of these common genes showed that enrichment scores >1.3 and *P-*values <0.01 were restricted to the terms, steroid metabolism/homeostasis, protein transport/localization and negative regulation of differentiation/neurogenesis.

A discrepancy between global changes in histone modification and local transcriptional effects has been noted in ES cells depleted in DPY30, a key component of the MLL2 histone methyltransferase complex [41]. *Dpy30* -/- cells showed minimal transcription changes, despite major global and local reductions in H3K4 methylation [41]. However, DPY30 depleted cells showed reduction or absence of expression of lineage-specific genes following RA treatment and compromised neuronal differentiation [41].

In quiescent human CD4+ T-cells, hyperacetylation induced by the inhibition of HDACs with a mixture of butyrate and TSA, occurred on only a minority of genes, primarily those that were transcriptionally active [21]. Among silent genes, a subset of less than 15% was found to be susceptible to hyperacetylation in the presence of HDACi. The great majority of this subset were marked by elevated levels of H3K4 methylation and could therefore be categorised as bivalent, poised genes [42,43]. Transcriptionally active genes are generally enriched in H3K4 methylation, particularly at their promoters [44], and it may be that levels of H3K4me3 are a more important determinant of sensitivity to HDACi than transcription per se. In contrast, Hezroni *et al*. [40] found only modest local changes in H3K9ac in ES cells treated with 0.5 mM VPA (less than 20% overall after 4 h treatment); there was variation between genes but no evidence that acetylation was selectively increased at regions classed as bivalent [40].

Our results illustrate how the *Hoxb* genes in ES cells are regulated by a combination of cluster-wide and locus-specific mechanisms. Thus, all *Hoxb* genes are kept in a silent state, at least in part, by Polycomb silencing complexes, being marked by a blanket of the Polycomb-associated histone modification H3K27me3 [45]. Similarly, all *Hoxb* genes are activated when differentiating cells are exposed to RA (see [46] and references therein). Remarkably, there is evidence that a binding site for RA receptor gamma in the *Hoxa1* upstream enhancer is a key player in cluster-wide activation of *Hoxb* genes [46]. Locus-specific effects became apparent when the time-course of the RA response was followed, with clear differences in the times at which individual *Hoxb* genes are maximally expressed, from day 3 to day 8 of differentiation. In contrast to *in vivo* data [30,47], in ES cells grown and RA-treated in culture, we found no clear correlation between the timing of maximal expression (transcript level) of individual *Hoxb* genes and their position in the cluster. The clear-cut and consistent differences in timing show that regulatory mechanisms acting at the level of individual loci, or groups of loci, can moderate the cluster-wide effect triggered by RA.

All *Hoxb* loci showed increased H3K9 acetylation in the presence of the HDACi, VPA. VPA consistently increased levels of H3K9ac at upstream, promoter and coding regions across the entire *Hoxb* cluster. H3K27ac and H3K4me3 both showed more modest, though occasionally significant, VPA-induced increases. Perhaps surprisingly, H4K8ac, a modification that shows a global increase after VPA, remained unchanged at all *Hoxb* loci tested. It is interesting that levels of H3K27me3 were consistently lower across *Hoxb* genes in VPA-treated cells, though the reductions were always small and statistically insignificant for any single locus. The HDACi-induced increase in H3K9ac at *Hoxb* loci shows that H3 acetate groups are turning over at these loci, which must therefore contain both HDACs and histone acetyltransferases (HATs). It will be interesting to identify these enzymes. In human T-cells, regions that were sensitive to HDACi-induced hyperacetylation have not always been found to be rich in HDACs, leading to the suggestion that the association of HDACs with such regions was transient and unstable [21]. As the specificity of HDACs for particular histone types tends to be low [9,48], it is likely that the absence of VPA-induced acetylation of certain H3 and H4 lysines across *Hoxb* loci reflects the absence of catalytically-active HATs of the necessary specificity, rather than variations in HDAC levels or types. A strong overlap has been noted in mouse ES cells between peaks of H3K9ac and the HAT p300 [40]. However, the significance of this is uncertain and much remains to be learned about the *in vivo* specificity of the various HAT complexes. Knock-out experiments have shown that depletion of CBP/p300 specifically reduces acetylation at H3K18 and H3K27, while GCN5 depletion selectively reduces H3K9ac [49].

It is interesting that only *Hoxb4* and *Hoxb7* showed a significant increase in transcription following VPA treatment of wild-type ES cells. As VPA treatment increased H3K9ac across *Hoxb4* and *Hoxb7*, and across *Hoxb1*, *2* and *5*, where transcription does not change, we can conclude that increased H3K9ac levels are not caused by increased transcription, nor are they sufficient, in themselves, to trigger transcription in all *Hoxb* genes. Of the modifications tested, only H3K27ac showed VPA-induced increases selectively at *Hoxb4* and *7*, thus correlating with changes in transcription. It has been suggested previously that H3K27ac might exert its effects by displacing, and mitigating the silencing effects of, H3K27me3 [50], and such a process is consistent with our data. The results so far also indicate that increased transcription of *Hoxb* genes in the presence of continued Polycomb repressor complex (PRC) silencing can only be induced at loci that are pre-marked by higher than average levels of H3K4me3, H3K9ac and H3K27ac. Perhaps these modifications are part of a permissive chromatin environment that facilitates VPA-induced transcription. Our results show that a high level of H3K4me3 is not, in itself, necessary for the VPA-sensitive chromatin state at all *Hoxb* loci, though it may play a locus-specific role, perhaps in facilitating the up-regulation of transcription in response to HDACi [21].

As expected, the absence of functional PRC complexes in *Eed*/*Ring1b* dKO cells led to increased *Hoxb* transcription. However, the increase varied from 8-fold and highly significant for *Hoxb7,* to small and insignificant for *Hoxb1* and *Hoxb5.* Interestingly, removal of Polycomb did not make the *Hoxb* genes more susceptible to the effects of HDACi-induced acetylation. With the exception of *Hoxb1*, treatment of dKO cells with VPA caused little further increase in *Hoxb* expression. *Hoxb1*, for which there was a relatively small (not significant) increase in transcription following PRC inactivation, showed a robust increase when dKO cells were treated with VPA. Collectively, these findings suggest that increased H3K9ac acetylation (and perhaps acetylation at other lysines not yet tested) induced by VPA can partially override PRC-mediated silencing of some *Hoxb* genes (particularly *Hoxb4* and *Hoxb7*), but causes no further increase in transcription in the absence of PRC. Thus, with the exception of *Hoxb1*, an increase in activating marks even in the absence of Polycomb-associated silencing, does not, in itself, induce transcription. The results emphasize that *Hoxb1* has regulatory features that distinguish it from other genes in the *Hoxb* cluster. It may be significant that chromatin opening, perhaps progressing 3′ to 5′ through the cluster, has been shown to be important for *Hox* gene activation [31,51]. If *Hoxb1* chromatin is selectively decondensed in PRC-deficient cells, it could be more sensitive to VPA-induced acetylation, perhaps because this facilitates the transient attachment of HATs and HDACs [21].

Gene silencing is a multi-layered process, involving the combinatorial actions of histone and DNA modifications, chromatin condensation, DNA binding proteins, small RNAs and the intra-nuclear location of genes and gene clusters. Gene activation involves stripping away some or all of these silencing components and may not be an all or nothing process. As we have seen, the *Hoxb* genes can be significantly up-regulated by treatment with VPA, or by removal of the Polycomb complex, though the level of activation is far below that seen following induction of differentiation and treatment with RA. In contrast, most genes in ES cells are insensitive to VPA treatment, and even within the *Hoxb* cluster, the response of *Hoxb1* sets it apart from the other *Hoxb* genes. Definitive conclusions about mechanisms of action of HDAC inhibitors are made difficult by the wide range of HDAC substrates, including both histones and non-histone proteins, but of the modifications tested, H3K9ac and H3K27ac are associated in both general and locus-specific ways with *Hoxb* regulation. These related modifications could provide both a context and the final trigger by which VPA activates *Hoxb* expression, and it is important now to define the regulatory pathways by which they are put in place and through which they operate.

## Conclusions

The HDACi, valproic acid, induces global histone hyperacetylation and increased H3K4 methylation in mouse ES cells, but significantly alters the expression of only a small proportion of genes, of which approximately one third are down-regulated.

There is no correlation between changes in expression and levels of H3K9ac, H4K8ac or H3K4me3 at gene promoters, which overall show little sign of VPA-induced change in histone modifications. It seems that most genes in mouse ES cells are sheltered from the global increases in histone modification induced by VPA.

*Hoxb* genes were exceptional in showing consistently increased H3K9ac at promoter and intragenic regions in cells exposed to VPA. H3K27ac and H3K4me3 showed smaller increases at only some *Hoxb* loci while H4K8ac was unaffected. VPA consistently caused small reductions in H3K27me3, though these never achieved statistical significance for any one locus. Only *Hoxb4* and *Hoxb7* showed significantly increased transcription after VPA treatment. These two loci have the highest levels of H3K9ac, H3K27ac and H3K4me3 in untreated cells and show strong increases in acetylation in response to VPA.

Inactivation of PRC in *Eed*/*Ring1b* dKO cells, activated *Hoxb* genes several-fold, with the exception of *Hoxb1* (only weakly activated) and *Hoxb5*, which remained essentially silent. Uniquely, *Hoxb1* activity in PRC-negative cells was increased several-fold by VPA. Thus, *Hoxb* genes respond to VPA in a Polycomb-dependent and locus-specific manner.

RA increased transcription of all *Hoxb* genes within 24 h, with an accompanying increase in H3K9ac, but expression thereafter, up to day 8, varied between *Hoxb* loci. In differentiating ES cells there is no clear relationship between the change in *Hoxb* expression over time and 3′ to 5′ position within the cluster.

Control of *Hoxb* genes is multilayered. Both PRC-mediated silencing, with high levels of H3K27me3, and RA activation, affect all *Hox* genes in the cluster. However, these overall control mechanisms are modulated in different ways at individual *Hoxb* loci. H3K9ac, H3K27ac and H3K4me3 may all help provide a VPA-responsive chromatin environment at specific *Hoxb* loci, but H4K8ac seems not to be involved.

## Methods

### Primary cells and cell culture

The male mouse feeder-independent 129/Sv-derived ES cell line, CCE/R [52], was grown on 0.1% gelatin-coated flasks and 129S4/SvJae-derived ES cell line, J1, and the *Eed*/*Ring1B* dKO, PRC1/PRC2-deficient) daughter cell line were grown on mitotically inactivated mouse embryonic fibroblast (MEF) feeder cells [39]. All ES cell lines were cultured in DMEM supplemented with 20% foetal calf serum, 1× penicillin/streptomycin, 1× L-glutamine, 1× non-essential amino acids, 0.25% 2-mercaptoethanol and 1U/μl ESGRO (Millipore, Watford, UK). All supplies were sourced from Invitrogen (Paisley, UK) unless otherwise noted.

For RA-induced differentiation, ES cells were plated on non-adherent plastic dishes in ES cell media minus ESGRO with addition of 1μM *all-trans*-RA on day 2 of differentiation [31]. Cell viability was assessed by Trypan Blue staining and by the MTS assay (Promega, Southampton, UK), a calorimetric assay to detect metabolically active cells (used according to the manufacturer’s instructions). ES pluripotency was tested by AP staining of undifferentiated fixed adherent cells [53,54]. Cell cycle analysis was performed using PI (10 μg/ml) staining in the presence of RNaseA (200 mg/ml) using a FACScan flow cytometer (BD FACSCalibur™, Becton Dickinson, Oxford, UK) and analysed using *WinMDi 2.9* and *Cylchred* software.

### Antibodies

Rabbit polyclonal antisera to H4K8ac, H3K9ac, H3K4me3 and H2BK12/15 ac were raised in-house by immunisation with synthetic peptides conjugated to ovalbumin as previously described [55]. Sources of other antisera are shown in Additional file 1: Table S4. Specificities were assayed by inhibition ELISA for all in-house and commercial antisera [55] and checked by western blotting.

### Protein analysis

Histones were extracted from ES cells by acid extraction and analysed by electrophoresis in 15% SDS-polyacrylamide gels and western blotting as previously described [1] Protein loading was tested by Ponceau S staining before proteins were probed with appropriate primary antibodies. Primary antibody binding was detected by fluorescent tagged anti-rabbit IgG secondary antibodies (Rockland) and detected by scanning (Odyssey system; LI-COR, Cambridge, UK).

### Native chromatin immunoprecipitation (NChIP)

Immunoprecipitation of native chromatin was performed based on the method described previously [56] and DNA was analysed using qPCR. Briefly, cells were lysed to release nuclei prior to micrococcal nuclease digestion. The amount of micrococcal nuclease added and digestion time were adjusted to obtain a mix of mono- and short oligo-nucleosomes, optimal for immunoprecipitation [56]. Digested chromatin was immunoprecipitated and bound (B) and unbound (UB) fractions were separated. DNA from B and UB fractions was isolated and analysed by qPCR using region specific primers. Sequences of all ChIP-qPCR primers are listed in Additional file 1: Tables S5-S7.

### Gene expression analysis

Total RNA was extracted from cells using the RNeasy mini kit (Qiagen, Crawley, UK) according to the manufacturer’s instruction. An on-column DNase digestion was performed during the extraction in order to yield a pure RNA fraction (RNase-Free DNase Set, Qiagen). cDNA was prepared using the RT-Superscript-III Kit (Invitrogen) according to manufacturer’s instructions. cDNA was then analysed using qPCR or microarrays.

For each RT-qPCR assay, 50 ng of cDNA were mixed with 5μl of SYBR green Mix (Qiagen), forward and reverse primers (0.4 μM each) and RNase-free water in a final volume of 10 μl. The PCR reaction was run on the ABI 7900HT system (95°C for 10 minutes; 40 cycles of 94°C for 15 s, primer set-specific annealing temperature for 30 s 72°C for 30 s, with a final denaturation step 95°C for 15 s, 60°C for 15 s, 95°C for 15 s). Standard curves made from serial dilutions of cDNA allowed relative quantification. Sequences of all primers and Tm used for qPCR are listed in Additional file 1: Table S8.

For microarray expression analysis we used the NIA 15 K mouse cDNA array [33] as previously described [34]. cDNA samples to be compared were labelled with two different fluorochromes, cy3 and cy5 (Amersham, Little Chalfont, UK) using a Bioprime Labelling kit (Invitrogen). Following their purification (PCR purification Kit; Qiagen), equal amounts of probes were combined and precipitated before hybridization onto slides at 42°C for 20 h. Slides were scanned using GenePix 4000A scanner. Spot alignment was checked automatically and then manually using GenePix software. Data were normalised using the DNMAD web-software. *T*-tests were performed on total M-values using biological triplicates for each condition and standard *P*-values were then corrected by the Benjamini-Hochberg FDR method using the statistical environment “R”. Finally, genes with associated FDR less than 10% were considered statistically significant and selected for further analysis. Original microarray data files are available through the GEO database [GSE45610].

## Abbreviations

AP: alkaline phosphatase; B: bound; ChIP: chromatin immunoprecipitation; CTCL: cutaneous T-cell lymphoma; dKO: double knockout (*Eed/Ring1b* negative); DMEM: Dulbecco’s modified Eagles medium; ELISA: enzyme-linked immunosorbent assay; ES: embryonic stem; FACS: fluorescence activated cell sorter; FDA: Food and Drug Administration; FDR: False Discovery Rate; HAT: histone acetyltransferase; HDAC: histone deacetylase; HDACi: histone deacetylase inhibitor; Hox: Homeobox; Ig: immunoglobulin; LIF: leukaemia inhibitory factor; MEF: mouse embryonic fibroblast; PI: propidium iodide; PRC: Polycomb repressor complex; RA: retinoic acid; RARE: retinoic acid response element; RT-qPCR: real time quantitative polymerase chain reaction; SAHA: suberoylanilide hydroxamic acid; SE: standard error; TSA: Trichostatin A; UB: unbound; VPA: valproic acid (administered as the sodium salt); WO: wash out.

## Competing interests

The authors declare that they have no competing interests.

## Authors’ contributions

EB carried out the bulk of the experimental work, conceived experiments, prepared figures and helped write the paper. HS carried out initial ES cell *Hox* gene experiments on which the present work is based. JAH contributed to experiments and helped write the paper. CER performed some ChIP experiments and helped write the paper. ML provided the dKO ES cells and advised on their use. AW provided the dKO ES cells and contributed to writing the paper. LPO advised on aspects of experimental work and helped write the paper. KPN advised on aspects of experimental work and helped write the paper. BMT conceived experiments, helped write the paper and coordinated assembly of the manuscript. All authors read and approved the final manuscript.

## Supplementary Material

Additional file 1 **Tables S1–S5. Table S1. **Classes of HDACs and HDAC inhibitors. **Table S2.** Functional annotation of genes whose expression is altered by VPA. **Table S3. **List of genes whose expression is altered by VPA in both the present experiments and those of Hezroni et al. [39]. **Table S4. **Source of antibodies. **Table S5. **ChIP-qPCR primers, *Hoxb1-13 *promoter regions. **Table S6. **ChIP-qPCR primers, *Hoxb1,2,4,5 and 7.***Table S7. **ChIP-qPCR primers, non-*Hox *genes. **Table S8. **RT-qPCR expression primers. Click here for file

Additional file 2 **Figures S1-S5. Figure S1. **Chromatin immunoprecipitation for H3K9ac, H3K4me3 at *Hoxb *promoters after 0, 2 and 8 h in valproic acid (VPA). **Figure S2. **Non-persistence of H3K9ac at *Hoxb *loci after removal of valproic acid (VPA). **Figure S3**, characterisation of Eed/Ring1b double knockout (dKO) cells. Click here for file
