# Supplementary material for: The histone deacetylase inhibitor sodium valproate causes limited transcriptional change in mouse embryonic stem cells but selectively overrides Polycomb-mediated Hoxb silencing
Source: Epigenetics Chromatin. 2013 May 1;6:11. doi: 10.1186/1756-8935-6-11 (PMC3769143; doi:10.1186/1756-8935-6-11)
Supplement: Additional file 1 — Tables S1–S5. Table S1. Classes of HDACs and HDAC inhibitors. Table S2. Functional annotation of genes whose expression is altered by VPA. Table S3. List of genes whose expression is altered by VPA in both the present experiments and those of Hezroni et al. [39]. Table S4. Source of antibodies. Table S5. ChIP-qPCR primers, Hoxb1-13 promoter regions. Table S6. ChIP-qPCR primers, Hoxb1,2,4,5 and 7.Table S7. ChIP-qPCR primers, non-Hox genes. Table S8. RT-qPCR expression primers. [file 1756-8935-6-11-S1.pdf]

ADDITIONAL FILE 1, TABLES

Additional Table S1
Structural classes of HDAC inhibitors in clinical trials or widely used experimentally.

Structural class	Name	HDACs inhibited	Clinical trial status
Hydroxamic acid	SAHA (Vorinostat)	I, IIa, IIb, IV*	FDA approved, (CTCL) [1]
	Panobinostat	I, II	I
	TSA	I, II	*Lab use only*
	At least 5 others	*various*	I/II
Short chain fatty acids	Vaproic acid	I, IIa	I
	Butyric acid	I, IIa	*Lab use only*
	AN9		II
Cyclic peptides	Depsipeptide (Romidepsin)	HDAC1,2,4,6	FDA approved, (CTCL) [1]
Benzamides	SNDX-275	HDAC1,2,3,9	II
	MGCD0103	HDAC1,2,3,11	II

* HDAC classes:
Class I, HDAC 1, 2, 3, 8
Class IIa, HDAC 4, 5, 7, 9
Class IIb, HDAC 6, 10
Class IV, HDAC 11
Class III, SIRT1-7 (NAD-dependent, not inhibited by HDACi)

REFERENCES

1. "StatBite: FDA oncology drug product approvals in 2009." J Natl Cancer Inst **102**(4): 219.

See main text for other references.

Additional Table S2

Functional annotations of genes found to shown changes in expression in response to VPA treatment, carried out with DAVID. An enrichment scores (E.S.) greater than or equal to 1.3 is significantly overrepresented in the dataset.

Cluster	E.S.	Examples of affected genes
Cytoskeleton/ cytoskeleton interracting proteins	4.29	*Actinβ, Arpc2, Coronin, Destrin, tropomyosin, coronin, destrin, gelsolin, vinculin, laminA.*
Transport	3.35	*Cldn6, Eea1, Dstn, Tcte1l, Ipo11, Kif3a, Kpnb1, Nsf, Sl4a2, Vdac1.*
Ubiquitine conjugation	3.31	*Nedd4, Psmb4, Rnf128, Mir, CathepsinL1, Smurf2.*
Cell cycle	3.17	*Ccnd1, Ccnd2, Ccne1, hcfc1, Mybl2, Anapc1.*
Vesicule formation	3.09	*Cltb, Ap2a1, Rab3d, Rad18, Scamp1, Itsn2.*
Lysosome metabolism	3.05	*Ctsl, Gm2a, Bcl10, Slc11a2, Manba.*
Transcription cofactors	2.41	*Nlk, Tbl1rx1, Trim28, Hcfc1, Ncoa1, Creg1, Maged1.*
Steroid/lipid metabolism	2.25	*Dpm1, Mvd, Pcyt1a, Lss, Agpat3.*
Chromatin organisation	2.19	*Dnmt3a, H1f0, H2afy2, H3f3b, Hira, Kdm1b-2a-3a-3b-6a, L3mbtl3, Myst2.*

Additional Table S3
List of genes whose expression is altered by VPA in both the present experiments and those of Hezroni et al [39].

Boudadi *et al.* (8hr 1mM VPA) vs. Hezroni *et al.* (4hr 0.5mM VPA, GSE23957)

Up

1600029D21Rik	Gclc	Ppfibp1
Akap11	Gldc	Ppp3ca
Ank	Gm1564	Prkcd
Arhgap18	Gpd1l	Prkcz
Arpc5	H1f0	Ptpn21
Atp8b2	Ifi30	Scarb1
Bcorl1	Inpp5f	Sec24d
Carhsp1	Klhdc2	Sh3bgrl
Cd24a	Lgals8	Sh3bgrl2
Cd47	Lpl	Slc25a30
Cdc14a	Lss	Soat1
Cldn6	Mak	Spg20
Creg1	Marcks	Stat2
Ctsl	Ndrg4	Stx7
Dhcr7	Nubp2	Sv2a
Dpysl2	Phf13	Tuft1
Ehd4	Pja2	Wwtr1
Fgd6	Pkp2	Ypel5

Down

Bnc2
Brd8
C77370
Ceacam1
Gdf3
Hcfc1
Hmgb1
Igf2bp1
Kpnb1
Mrps18b
Nanog
Nr5a2
Ntn1
Pprc1
Sall4
Trh
Ywhah
Zfp57

Boudadi et al. vs. Hezroni et al. (16hr 0.5mM VPA, GSE23956)

Up	Down
6430548M08Rik	8430406I07Rik
Akap11	Ccnd1
Arhgap18	D17H6S56E-5
Cd24a	Etv5
Cldn6	Gdf3
Clu	Gjb3
Cpeb1	Gli2
Eea1	Gtf2i
Egr1	Hmgb1
Gm1564	Igf2bp1
H1f0	Mllt10
Ndrg4	Tbx3
Nucb2	Zcchc10
Pik3cd	Zfp57
Sec24d	

Additional Table S4

Details of antibodies used in the study. All antisera with R numbers have been made and characterised in-house.

Antibody	Raised against
R403	H4K8ac
R41	H4K5ac
R252	H4K16ac
R607	H3K9ac
R612	H3K4me3
Millipore 07-449	H3K27me3
Ab-4729	H3K27ac
R209	H2BK12,15ac
Ab-1791	H3 C-term
Ab-19857	Oct4
Ab-26648	Klf4
Ab-8227	Actin
Rockland 611-131-122	Rabbit IgG
Rockland 611-131-121	Mouse IgG

Additional Table S5

ChIP-qPCR primer sequences for analysis of Hoxb promoter regions

	Forward Primer	Reverse Primer	Amplicon Length
Hoxb1	TTCCATGTCGCTCTCAGATG	TAGAAGGGGCTAGGGAGTG	110
Hoxb2	GCTTTGTCTTGGTTGTGGTG	TCCAAGGAGCAGCTTTGACT	139
Hoxb4	GCTAAATGCGATTTGGAAGG	GAGGGGTAGGAGGTGAGTGA	147
Hoxb5	ACGACTGGTCAACAAAAGCA	GCGATGCACTCTACTTCGTT	112
Hoxb6	ATTTCCTTCTGGACCCTCACT	GCCGGGTTTATGATTTGTTG	119
Hoxb7	GTAAATGCGAGCAGGAGGAG	AGTCCCAGCTGGAGCCTACT	110
Hoxb8	CAACAACAGACTCCGGCTTT	TATCGTGTGGAGGGAATTGG	100
Hoxb9	TCCAAAGCCCTCTGCAC	GACATTCTCACACATTATCCGCG	10
Hoxb13	GTGCGGGAGGGATTTTATGAG	GTTTTAAATCGCTCCCAGCTC	136

Additional Table S6
ChIP-qPCR primer sequences and annealing temperatures for analysis of Hoxb genes

	Forward primer	Reverse primer	Annealing temp (°C)
Hoxb1/1	AGCCCTACAGCCTTGGGGTGG	TTCAACCCAGCTGCCCCTCAC	58
Hoxb1/2	TCCTAGGGGATCGCTGGCGG	GCCGTTTGGGGGAGACACCC	58
Hoxb1/3	CCGCAGCCCCCATACGGAAC	CGGGGAGTGAGAGTGCTGGGT	60
Hoxb2/1	TCCCCCAAGGTATGGAAGCCTCT	CCTACCACCACCTCAGCCCCC	60
Hoxb2/3	CAAGCCGGAGATGGGCCTGC	GAGGCGGCTGGAGAAGGGGA	60
Hoxb4/1	GCTACACCCGCAGCCCCAAC	GAGACCCCCTCCTCGCCTGG	60
Hoxb4/2	GGTGGCCATTGGCTCGGAGG	CGCACGGAGGGAACTTGGGG	60
Hoxb4/3	TGTCCCCTCGGGCTCCAGTG	GGGCTCTTTGCACGCGGAGT	60
Hoxb5/1	AGAGGGGATAGCCGCACCCT	CTGGCTCTGTGCAACCGCCA	58
Hoxb5/2	TGGCCGCATACATAGCAAAACGA	GGAGGGCTTGATTTGTGGATCGTGG	58
Hoxb5/3	GCCCTGCACTAACGGCGACA	TCCTCGGGCTCAGAGGACGC	60
Hoxb7/1	CACCAAGTTGCGAACATTCA	CAGCTTGCAGGCGAGATT	60
Hoxb7/2	ACCGACACTAAAACGTCCCCGA	AGCGAAAACCGAACTTGCGGC	58
Hoxb7/3	GCGCCAAGGAGCAGAGGGAC	CGGAGCCCTTCTGGGGACCG	58

Additional Table S7

ChIP-qPCR primer sequences and annealing temperatures for analysis of genes that were found to show distinct transcriptional responses to VPA in ES cells.

Gene	**Forward primer**	**Reverse primer**	**Location**
Egr1/1	GGCAAGCTGGGAACTCCA	ACCCGGAGTGACGTGAAG	-260
Egr1/2	GGCTTTCTGCTTCCCATA	TGGGATCTCTCGCGACTC	-97
Egr1/3	CTGCACCCCGCATGTAAC	GCGAGCTGGAGAACTGATGT	+173
Egr1/4	TGTTGTTTCTCCTGGGCTTG	AGTTCCCAGAACCACCACAC	+4336(in.2)
H1f0/1	GCCCTTCCAACTCTTTCAGG	CGGCCCCAACTAACACAC	-380
H1f0/2	GAGTGGGGCTCCTGGTAGTC	GGCACATTTCCCCTTTGC	-90
H1f0/3	GGTGCGTTTTTCTTTAACTGG	TCTCCCGAAGACCCACCTAC	+2870 (in.1)
Ndrg4/1	AGCTTCTCCTCGGGTCTAGC	GCACCATCCATTAGGTACAGC	-425
Ndrg4/2	CTTGAGCGGCAGAAGCTG	CCTCCCTGCAAGGTTTCTG	-250
Ndrg4/3	GAGCTCTACCCCACCACAGA	GACCCTCACTCACCCCATC	-24
Ndrg4/4	CCTATCACAGGGAAGGCTCA	CAGCCTGGGCTACAGAAAGA	+12558 (in.15)
Nanog/1	GCAGCCGTGGTTAAAAGATG	AGGGAGGGAAAGCTTAGGG	-182
Nanog/2	ATCCACTGAGCCCATCTCACC	CTGGTCTGCAGAGCTAGTTCAA	+6222 (in.5)

All primer pairs annealed at 60°C. All primers were adjacent to the TSS except where shown as intragenic (in.2 etc).

Additional Table S8

RT-qPCR primer sequences and annealing temperatures.

	Forward primer	Reverse primer	Annealing temp (°C)
β-actin	CGTGAAAAGATGACCCAGATCA	TGGTACGACCAGAGGCATACAG	58
Gapdh	CGGCCGCATCTTCTTGTGCA	AGTGGGGTCTCGCTCCTGGA	54
Oct4/pouf5	GAGGAGTCCCAGGACATGAA	AGATGGTGGTCTGGCTGAAC	60
nanog	CTCATCAATGCCTGCAGTTTTTCA	CTCCTCAGGGCCCTTGTCAGC	62
klf4	CCAGACCAGATGCAGTCACAA	TGGCATGAGCTCTTGATAATGG	62
c-Myc	AGCAGCTCGAATTTCTTCCA	AGAGCTCCTCGAGCTGTTTG	60
Sox2	GCGGCAACCAGAAGAACAG	GTTGTGCATCTTGGGGTTCT	58
H1f0	ACCATGACCGAGAACTCCACCTC	GTGGTCACTAGGCGCTTGAT	58
Egr1	GAGCGAACAACCCTATGAGC	AGGCCACTGACTAGGCTGAA	60
Ndrg4	CCTACCTCCAGACCGAGTGA	GAAGTCCATAAGGCGTCTCG	60
Fsd1	AAGGCTAGTTGGCTGCTGTC	TCAGCAACATCTGCTTCAGG	62
Dnmt3	GGCAATTCCTTCTCTGAAGC	ACGGTTCTCCTCCTGTTCCT	60
Pgk1	AATGTCGCTTTCCAACAAGC	TTTGATGCTTGGAACAGCAG	60
Gdf3	TCAGCTTCTCCCAGACCAGGGTTTT	GCCCCGGTCCTGAACCACAG	60
Nupr1	ACAGCCTGGCCCATCCCTGT	CTCCAGCTCCGTCTCAGCGC	60
Pprc1	GGAGGCGGCGGTACAGCTCT	CTCGTCTGCCTGCCGCAACT	58
Pnrc2	GCGGCCTCCATTTTGTCGGG	ACAGCCCACGAACGAGTCACG	58
Slc6a8	GAGACTTGGACACGCCAGAT	AAAATGGGGATTCCTCCAAC	58
Anapc7	GAGTACCGCAATGCTGTGAG	TCGAGTACAGCAATGGCATC	55
Hoxb1	CCATATCCTCCGCCGCAG	CGGACTGGTCAGAGGCATC	58
Hoxb2	CGGCGCCTCCACCCTTCAGAGACC	CTTTCGGTGAGGTCCAGCAAGGC	60
Hoxb3	CAACTCCACCCTCACCAAA	GCCACCACCACAACCTTC	58
Hoxb4	CTGGATGCGCAAAGTTCAC	TCCTTCTCCAACTCCAGGAC	62
Hoxb5	CCTCTGAGCCCGAGGAAG	CCAGGGTCTGGTAGCGAGTA	60
Hoxb6	GAGACCGAGGAGCAGAAGTG	ACTGAGCTGAGACGCACTGA	62
Hoxb7	AACCGAGTTCCTTCAACATG	CGAGTCAGGTAGCGATTGTA	55
Hoxb8	TTCTACGGCTACGACCCTCT	CGTGCGATACCTCGATCCTC	60
Hoxb9	CAGGGAGGCTGTCCTGTCTAATC	CTTCTCTAGCTCCAGCGTCTGG	58
Eed	TGATTGTGTGCGATGGTTAGGCG	GCCCAATGCAAGCATCTTTTGCCA	60
Rnf2/Ring1b	CTGGGGCAGGAGCCGAAATGTC	TCCGCGCAAAACCGATGTAAACAC	60
