# Supplementary material for: The histone deacetylase inhibitor sodium valproate causes limited transcriptional change in mouse embryonic stem cells but selectively overrides Polycomb-mediated Hoxb silencing
Source: Epigenetics Chromatin. 2013 May 1;6:11. doi: 10.1186/1756-8935-6-11 (PMC3769143; doi:10.1186/1756-8935-6-11)
Supplement: Additional file 2 — Figures S1-S5. Figure S1. Chromatin immunoprecipitation for H3K9ac, H3K4me3 at Hoxb promoters after 0, 2 and 8 h in valproic acid (VPA). Figure S2. Non-persistence of H3K9ac at Hoxb loci after removal of valproic acid (VPA). Figure S3, characterisation of Eed/Ring1b double knockout (dKO) cells. [file 1756-8935-6-11-S2.pdf]

ADDITIONAL FILE 2, FIGURES

Additional Figure S1

A

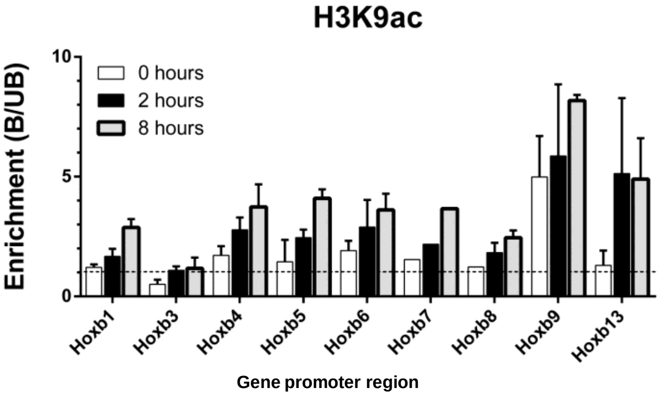

B

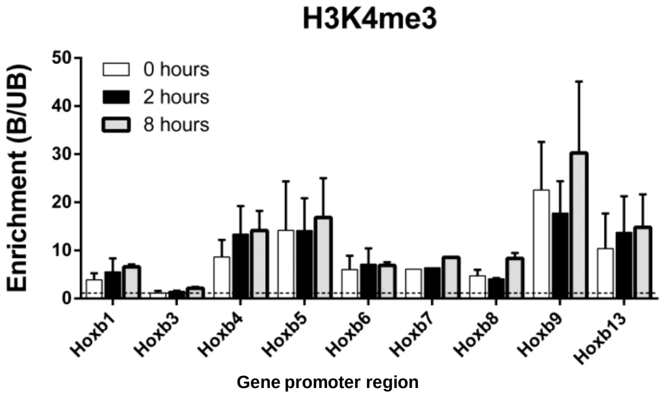

C

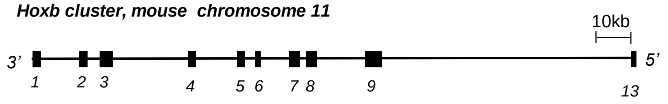

VPA progressively increases H3K9ac but not H3K4me3, at Hoxb promoters in mouse ES cells

(A, B) Undifferentiated CCE/R ES cells were treated with 1mM VPA for 0, 2 and 8h. Chromatin was extracted and immunoprecipitated with antibodies to H3K9ac (A) and H3K4me3 (B). DNA corresponding to each *Hoxb* promoter was quantified by RT-qPCR using primers listed in Table 5, Additional File 1. PCR was performed in triplicate from each of two independent experiments, except for *Hoxb7*, for which only one experiment was possible. When these experiments were performed, we had been unable to prepare adequate primers for *Hoxb2*. Results are expressed as the amount of gene-specific DNA in the antibody bound fraction relative to the input fraction (Bound/Input). Values above 1.0 (dashed line) represent enrichment relative to bulk chromatin. Error bars represent the standard error of the mean.

(C) Spacing of individual *Hoxb* genes across the cluster.

Additional Figure S2

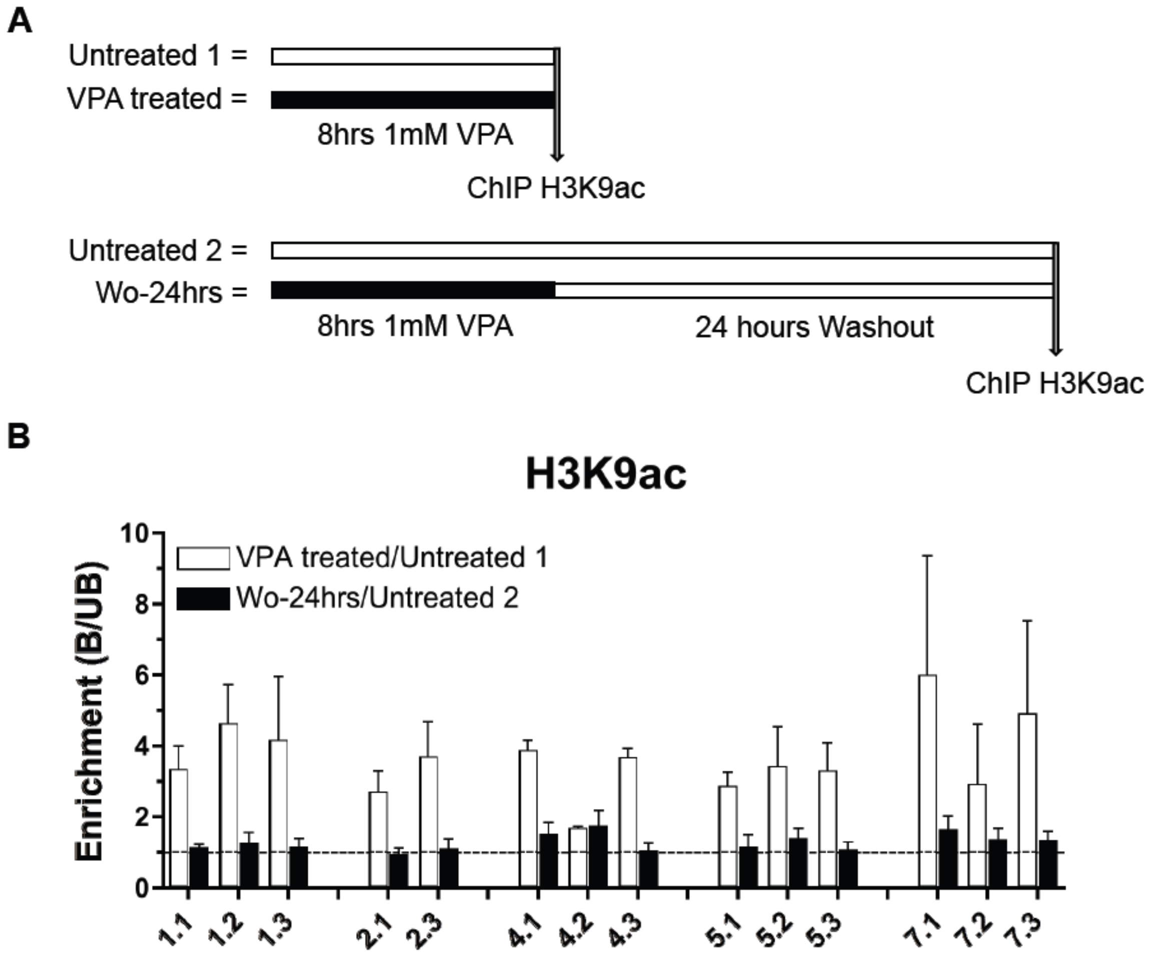

In ES cells, VPA-induced increases in H3K9ac at Hoxb promoters do not persist through mitosis in the absence of inhibitor

A. Design of the experiment. ES cells grown for 8h in 1mM VPA and untreated controls were harvested, chromatin was prepared and immunoprecipitated with antibodies to H3K9ac. A second batch of cells was treated for 8h with 1mM VPA then washed and returned to culture in the absence of VPA. After 24h the washout (Wo) cells were harvested, along with untreated controls, prior to chromatin preparation and H3K9ac immunoprecipitation (ChIP).
B. Results of ChIP experiments showing levels of H3K9ac at two or three sites across five *Hoxb* genes immediately after VPA treatment and after 24h washout. Results are presented as the H3K9ac levels at each site in VPA-treated cells relative to the levels in untreated control cells harvested in parallel. After 8h in VPA H3K9ac levels increased by up to three fold at each site (pale bars) but were indistinguishable from control values after 24h in the absence of VPA (dark bars).

Additional Figure S3

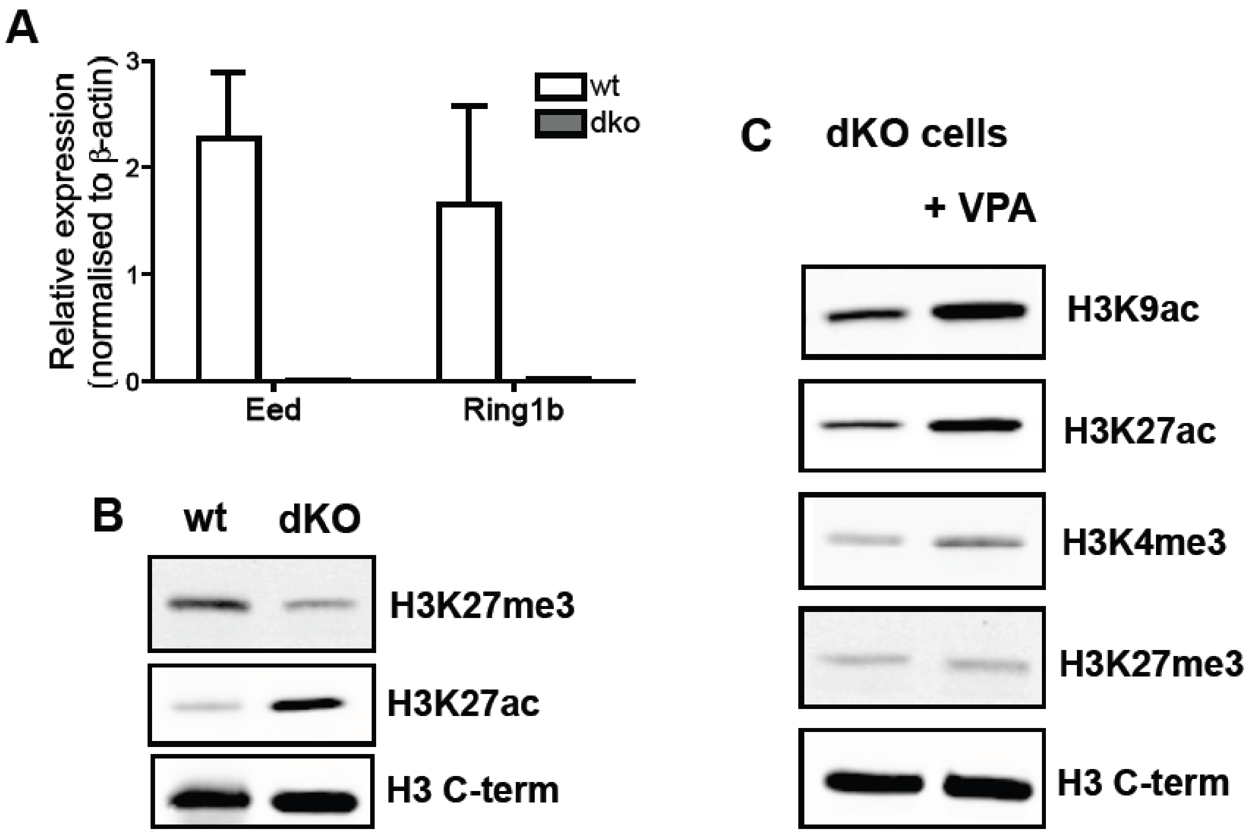

Characterisation of double knockout (dKO) ES cells.

A. Quantification by RT-qPCR of *Eed* and *Ring1b* transcripts in wild type (J1 line) and in dKO ES cells. Transcripts were undetectable to dKO cells. Error bars represent the standard error of the mean of independent experiments (n=3).

B. Bulk histones were extracted from wild type and dKO ES cells and levels of H3K27me3 and H3K27ac were assessed by western blotting. Antibody binding to the unmodified H3 C-terminal region was used as a loading control.

C. **Bulk histones were extracted from dKO ES cells before and after treatment with VPA (1mM, 8h). Western blotting was used to visualise changes in H3K9ac, H3K27ac, H3K27me3 and H3K4me3). H3 C-term was used as a loading control.**
